# Rethinking alcohol interventions in health care: a thematic meeting of the International Network on Brief Interventions for Alcohol & Other Drugs (INEBRIA)

**DOI:** 10.1186/s13722-017-0079-8

**Published:** 2017-05-10

**Authors:** Joseph E. Glass, Sven Andréasson, Katharine A. Bradley, Sara Wallhed Finn, Emily C. Williams, Ann-Sofie Bakshi, Antoni Gual, Nick Heather, Marcela Tiburcio Sainz, Vivek Benegal, Richard Saitz

**Affiliations:** 1 Kaiser Permanente Washington Health Research Institute, Kaiser Foundation Health Plan of Washington, 1730 Minor Avenue, Suite 1600, Seattle, WA 98101 USA; 2 Department of Public Health Sciences, Karolinska Institutet, Stockholm, Sweden; 3 Health Services Research & Development (HSR&D), Center of Innovation for Veteran-Centered and Value-Driven Care, Veterans Affairs (VA) Puget Sound Health Care System, Seattle, WA USA; 4 Department of Health Services, University of Washington, Seattle, WA USA; 5 Centre for Psychiatry Research, Department of Clinical Neuroscience, Karolinska Institutet & Stockholm Health Care Services, Stockholm, Sweden; 6 Addictions Unit, Psychiatry Department, ICN, Hospital Clínic, IDIBAPS, RTA, Barcelona, Spain; 7 Department of Psychology, Faculty of Health & Social Sciences, Northumbria University, Newcastle upon Tyne, UK; 8 Department of Social Sciences in Health, Ramón de la Fuente Muñiz, National Institute of Psychiatry, Mexico City, Mexico; 9 Centre for Addiction Medicine, National Institute of Mental Health and Neurosciences, Bangalore, India; 10 Department of Community Health Sciences, Boston University School of Public Health, Boston, MA USA; 11 Clinical Addiction Research and Education Unit, Section of General Internal Medicine, Boston Medical Center and Boston University School of Medicine, Boston, MA USA

**Keywords:** Alcohol, Hazardous alcohol use, Harmful alcohol use, Alcohol dependence, Screening, Brief intervention, Referral to treatment, Research agenda, Health care, International

## Abstract

In 2016, the International Network on Brief Interventions for Alcohol & Other Drugs convened a meeting titled “Rethinking alcohol interventions in health care”. The aims of the meeting were to synthesize recent evidence about screening and brief intervention and to set directions for research, practice, and policy in light of this evidence. Screening and brief intervention is efficacious in reducing self-reported alcohol consumption for some with unhealthy alcohol use, but there are gaps in evidence for its effectiveness. Because screening and brief intervention is not known to be efficacious for individuals with more severe unhealthy alcohol use, recent data showing the lack of evidence for referral to treatment as part of screening and brief intervention are alarming. While screening and brief intervention was designed to be a population-based approach, its reach is limited. Implementation in real world care also remains a challenge. This report summarizes practice, research, and policy recommendations and key research developments from our meeting. In order to move the field forward, a research agenda was proposed to (1) address evidence gaps in screening, brief intervention, and referral to treatment, (2) develop innovations to address severe unhealthy alcohol use within primary care, (3) describe the stigma of unhealthy alcohol use, which obstructs progress in prevention and treatment, (4) reconsider existing conceptualizations of unhealthy alcohol use that may influence health care, and (5) identify efforts needed to improve the capacity for addressing unhealthy alcohol consumption in all world regions.

## Background

The identification and management of unhealthy alcohol use [1] in general healthcare settings has been an international public health priority for decades [2–4]. Unhealthy alcohol use is a spectrum of drinking that includes drinking amounts that increase the risk of health harms (e.g., risky, at-risk or hazardous), drinking that has already caused harm (e.g., harmful), or a loss of control of drinking (known as alcohol dependence in the International Classification of Diseases (ICD) and in Diagnostic and Statistical Manual of Mental Disorders (DSM)-IV or moderate to severe alcohol use disorder in DSM-5) [1, 5, 6].

In May 2016, the International Network on Brief Interventions for Alcohol & Other Drugs (INEBRIA) convened a meeting titled “Rethinking alcohol interventions in health care” in Stockholm, Sweden to synthesize key developments in the management of unhealthy alcohol use in primary care/general practice settings and to set directions for future research in light of this evidence.

### Context: screening and brief intervention

Screening and brief intervention (SBI) is widely recommended for the prevention and early intervention of unhealthy alcohol use [7] based on evidence from randomized controlled trials conducted in many countries [8]. SBI consists of alcohol screening (often universal, or at least systematic) with a validated instrument [9, 10] followed by advice or brief intervention for those who exceed recommended drinking limits [11]. SBI is intentionally brief and limited in scope to maximize its feasibility as a population-based intervention in primary care. Consequently, it was not designed for severe forms of unhealthy alcohol use and is not known to be efficacious in these patients [12], although further study could find otherwise, particularly if repeated contacts are included [13, 14].

Based in part on research testing SBI for hazardous and harmful alcohol use in outpatient primary care settings [3], Screening, Brief Intervention, and Referral to Treatment (SBIRT) was developed in 2003 in the US for health care settings to address the full spectrum of unhealthy alcohol (and drug) use, including those with more severe alcohol-related conditions [15, 16]. Referral to specialty treatment, or the “RT” of SBIRT, would address SBI’s efficacy gap in helping those with severe problems, given that more intensive evidence-based treatments delivered by addiction specialists can be effective in those with severe conditions who seek help [17–23]. Professional organizations and national agencies have recommended or provided substantial funding for SBIRT in primary care [24–26].

In light of recent evidence, SBI is in need of new direction. The efficacy of SBI is limited to a narrow range of individuals without severe conditions [27]. The RT of SBIRT is not known to be efficacious in linking those with severe alcohol-related conditions to more specialized or intensive treatments [28], raising concerns about the effectiveness of SBIRT for the full spectrum of unhealthy alcohol use. The implementation of SBI in primary care remains a challenge [29–33], in part because clinicians are reluctant to initiate discussions about alcohol owing in part to practical constraints, alcohol-related stigma, and uncertainty about how to assist patients with more severe alcohol problems [30, 31, 34, 35]. Yet, failing to identify and address unhealthy alcohol use, a leading cause of preventable death worldwide, seems an untenable course [36].

At the INEBRIA thematic meeting, which took place in Stockholm, Sweden, international experts were invited to review evidence and define a research agenda to (1) close evidence gaps in screening, brief intervention, and referral to treatment, (2) develop innovations to address more severe forms of alcohol consumption within primary care, (3) describe the stigma of unhealthy alcohol use, which obstructs progress in prevention and treatment, (4) reconsider existing conceptualizations of unhealthy alcohol use that may influence health care, and (5) identify efforts needed to improve the capacity for addressing unhealthy alcohol consumption in all world regions. Key developments from this meeting are described below by those who presented reviews.

### The impact of screening and brief intervention on outcomes

#### Richard Saitz, MD, MPH

Randomized controlled trials with clinically important outcomes are the types of studies needed to understand the true effects of alcohol-related interventions. Efficacy studies suggest that brief intervention in general practice/primary care decreases self-reported alcohol consumption among adults identified by screening [8]. However, important evidence gaps exist regarding the efficacy of SBI among women, younger and older persons, minority ethnic groups, those with comorbid mental health and drug use conditions, and those living in developing countries [27]. Evidence for improving important health outcomes, such as liver blood tests, alcohol-related diseases and injuries, healthcare utilization, costs, and death, is inconsistent [8, 37–40]. There are specific studies that find effects on some of these outcomes but they are not borne out in systematic reviews. Favorable conclusions about costs and utilization in the literature are generally based on individual trial results and on simulation models that use estimates from such studies in lieu of robust findings across trials. And they rely on assumptions about self-reported decreases leading to benefits in harder outcomes. The lack of consistent effects of SBI on “hard” outcomes raises questions about whether effects on consumption are real. Effects on consumption are seen in patients who are counseled to drink less and are then asked later if they are drinking less. In this setting patient responses could reflect social desirability bias; with no consistent effects on other outcomes, it remains possible that SBI has no efficacy for reducing consumption.

The effect of brief intervention on reducing alcohol consumption if real, is small [41–43], but small effects can be large across populations. To achieve those effects requires broad reach of valid screening and brief interventions with fidelity, which is unlikely owing to implementation barriers [44] and to the levels of skill needed to do SBI well. Policy recommendations often lead to screening, but screening poorly done [29], and policy recommendations regarding brief interventions focus on a single session or quick advice (whereas the best evidence for efficacy is for repeated BI [8]. Brief intervention requires skill and is difficult to learn and maintain. Implementing SBI universally is a huge effort and investment of training, systems, time and effort. Consequently, following implementation, effects on drinking and consequences are not always detected in real world practice [45, 46]. Several well-done studies of brief intervention implementation and effectiveness show it does not get implemented well (e.g., low rates of intervention) [47] or does not retain effectiveness, or both [32, 33, 45]. Electronic SBI might help solve some implementation challenges though not necessarily efficacy questions (small effects on self-reported drinking are observed) [48, 49].

What should we do with this evidence? Current health policy and practice recommendations may therefore go beyond the evidence for SBI. If efficacy or effectiveness is questioned, then cost and potential harm (of BI done poorly) need to be considered as well. We should question the current evidence and demand high quality randomized controlled trials with clinically important outcomes that are meaningful to patients. We should identify ingredients of current approaches that do and do not contribute to efficacy, and discover more efficacious interventions. Such interventions may involve repeating brief interventions over time. We should also work to identify any harms of SBI. This work could explore and mitigate detrimental effects of recording alcohol use disorders in medical records, which could affect insurability and employment.

While the field pursues this critical research, here are a few recommendations for policy and practice. We should not confuse alcohol SBI—a universal preventive service intended to reduce risks and harms—with other reasons and ways to identify and address unhealthy alcohol use. People should be informed about the true risks of use, for example pre-pregnancy or when prescribing a new medications, before driving, and the like. Clinicians need to know if their patients are drinking if they are going to properly diagnose just about any symptom (for example, anxiety, depression, high blood pressure, heartburn) or prescribe any medication. Screening tools designed for SBI do not necessarily provide this information. For example, the AUDIT-C, one of the best validated tools for use in SBI, asks about past-year drinking frequency [9]. The result of the test is sensitive and specific for past year unhealthy use. However, it is possible to have recent drinking of relevance to prescribing or to a symptom (e.g., heartburn) and not score in the unhealthy use range. It is also possible to have been abstinent, for months, despite affirmative responses to the questionnaire. Therefore, in practice it makes sense to ask clinically relevant and necessary questions for the task at hand (e.g., have you had any alcohol—beer, wine or liquor—today?) just as one would for other exposures such as medications, natural products, and diet. This approach as part of screening with a validated tool for use in SBI (and recommended for over a decade by the National Institute on Alcohol Abuse and Alcoholism) [50, 51] can identify both the needed clinical information for medical diagnosis and treatment, and what is needed to detect unhealthy use. When unhealthy use is identified (either by screening or in the course of a patient discussion), feedback, information and counsel should be provided, but we should not expect much in the way of changes in consumption or consequences. For those with more severe unhealthy use, there is no evidence for SBI efficacy; clinicians need to address that use with either longitudinal integrated care or referral to a specialist (see next section). For policy, if recommending universal SBI, the rationale could be to improve attention to alcohol in general and even to support staff who can facilitate getting help to those with more severe conditions who need and want it, but we should not expect BI to reduce alcohol consequences by itself. Health systems should recognize it will be costly and will require major implementation efforts and seriously reconsider whether tying SBI to quality measures that lead to withholding payments are appropriate (they are likely not).

### Referral to treatment

#### Joseph E. Glass, Ph.D., MSW

For SBIRT to achieve its goal to address all forms of unhealthy alcohol use, patients with alcohol use disorders would need to be linked to effective services such as intensive specialty treatment programs offering evidence-based treatments and mutual help programs available in the community [15, 52]. Referral is a common practice in primary health care, but referring patients with alcohol use disorders to specialty services may be more difficult because many do not believe that they have a problem or a need for treatment [53–55]. Among those who believe that they need help, they often report that they have reservations about seeking treatment (e.g., wanting to stop drinking on their own, not being ready to quit) [56–58].

Intuitively, referrals to specialty care could be more effective when delivered in the context of a motivational intervention delivered by a trained clinician [59, 60]. When SBIRT was formalized, RT was presumed to be efficacious in linking individuals to specialty addiction services based on this intuition and from observational research and controlled trials, even though these innovative trials lacked sufficient methodological rigor [15, 28, 61–66]. Another assumption was that patients identified opportunistically in primary care—a population different from treatment-seeking patients—would experience clinical benefit if they completed the referral and initiated specialty treatment. However, prior to deeming SBIRT as an evidence-based program, and prior to national agencies disseminating and providing significant funding for it, the efficacy of RT interventions on treatment utilization and clinical outcomes was not systematically evaluated.

Thus, a systematic review and meta-analysis of randomized controlled trials was conducted to evaluate the efficacy of RT [28]. Eligible studies were trials of brief alcohol interventions in medical settings including adult and adolescent samples that reported alcohol services utilization as an outcome. The study compared alcohol services utilization across intervention and control groups. Of fifteen eligible trials, eleven could be meta-analyzed (*n* = 1183 in intervention groups and *n* = 1197 in control groups). The main findings were not statistically significant; the pooled risk ratio was RR = 1.16 (95% CI 0.96–1.40), and no a priori subgroup analyses (stratified by age, setting, severity, risk of bias) yielded statistically significant results. Moreover, the identified studies did not provide sufficient data to examine an association between post-SBI treatment utilization and clinical outcomes. In a subsequent editorial the authors re-analyzed these studies along with additional trial data and found the same results [67].

Hence, a synthesis of randomized controlled trials points to a lack of efficacy of brief alcohol interventions, as currently implemented, in increasing alcohol service utilization via RT [28]. However, of studies on RT to date, the most promising trial demonstrated the possibility that administering brief alcohol interventions over multiple sessions could improve referral success [68], but this study needs replication given that other studies with multiple sessions did not find statistically significant effects of RT in promoting treatment linkage [28].

What should we do with this information? Because patients receiving SBI are identified opportunistically, a brief motivational intervention is probably too brief to help most people develop sufficient motivation to seek treatment [69–71]—a process that may be elusive owing to stigma and other barriers to treatment [72–75]. Rather, a more sensible approach would be for patients identified by screening to be offered repeated brief interventions over time within primary care with their response monitored and interventions adjusted as needed. Payers and professional organizations have emphasized behavioral health integration, which offers a mechanism for integrating care for unhealthy alcohol use into primary care settings, using approaches such as care management [76–78]. If proven effective, the provision of more intensive treatments for alcohol use disorders within primary care would substantially decrease the need for referrals to specialty treatment. Several promising interventions for alcohol use disorders in primary care have been evaluated in randomized controlled trials [79, 80], and new interventions (such as two novel approaches described in the following sections) are being rigorously evaluated. Nonetheless, it is likely that not all patients can receive the maximum benefit from care that be provided in primary care settings. Some patients may gain more benefit from treatments that would be optimally delivered in a specialty setting, such as time-intensive evidence-based interventions for patients with extensive psychosocial needs and the most severe alcohol use disorders [81–83]. Thus, it is critical that the field continues to develop and test RT interventions to help ease the transition from primary care to specialty treatment for patients who need and want it, while concurrently developing interventions for the primary care setting that could help the vast majority of patients with alcohol use disorders. However, the implementation of evidence-based interventions in real-world specialty treatment settings is limited, which makes it even more important to develop effective treatments for alcohol use disorders in primary care.

### Context: novel approaches to address alcohol use disorders in general healthcare

New treatment approaches are needed in primary care in order to provide effective services for alcohol use disorders to more people. Of the psychiatric disorders, alcohol use disorders have the largest gap between the number of people affected and the number in treatment. Fewer than one in ten individuals with past-year alcohol use disorder receive help [84, 85]. Numerous practical barriers exist to receiving treatment, and attitudinal barriers such as the stigma of unhealthy alcohol use and its treatment are a particular impediment [86].

In healthcare systems, an alternative approach to referring patients and hoping that they seek specialty care involves a greater role for primary care in the treatment of more severe alcohol use disorders. However, alcohol use disorders in primary care often go unrecognized. When alcohol use disorders are identified, individuals are typically advised to stop drinking or are referred to specialist treatment, but as noted above, most patients do not follow these recommendations. They might not think they have a problem with drinking, and even if they do, they might not think it is severe enough to have to stop drinking altogether and attend a (stigmatized) treatment. Experts have called for new care models to manage alcohol use disorders, including severe alcohol use disorders, in primary care. Two novel treatment models were presented at this thematic meeting.

### The CHOICE model of care for alcohol use disorders in medical settings: early qualitative reports

#### Katharine A. Bradley, MD, MPH

To respond to the need to develop effective treatments for alcohol use disorders in primary care settings, we developed the Choosing Healthier drinking Options In primary CarE (CHOICE) trial. CHOICE was a trial of nurse collaborative care for alcohol use disorders in US Veterans who received primary care at one of 3 U.S. Department of Veterans Affairs (VA) facilities. The CHOICE trial addressed a number of principles outlined in the prior sections, such as following patients longitudinally with repeated brief interventions and ongoing monitoring, providing medications for alcohol use disorders within primary care, and linking patients who wanted specialty treatment to the appropriate program. The design of the CHOICE intervention was informed by: collaborative care models shown effective for depression and other chronic conditions [87] and based on Wagner’s Chronic Care Model [88] and patient-centered primary care including shared decision-making [89]. The 12-months CHOICE intervention was offered by registered nurses. The intervention included an engagement visit, repeated brief interventions using motivational interviewing skills and shared decision-making regarding treatment options including self-assessment, self-monitoring (with or without laboratory biomarkers), and alcohol use disorders medications. A study nurse practitioner was available to prescribe and manage medications for alcohol use disorders. All CHOICE nurses were supervised in weekly collaborative care team meetings by an interdisciplinary team including: two psychologists, two addiction psychiatrists and two general internists. CHOICE can be set apart from other similar recent randomized controlled trials targeting alcohol use disorders in primary care [79, 80] because of its combination of several unique characteristics: it targeted individuals at high risk for alcohol use disorders who were already engaged in primary care, it included individuals regardless of their comorbidity, and it intervened only through the addition of a collaborative nurse care manager and primary care nurse practitioner prescriber of medications supported by interdisciplinary consultants.

Because alcohol use disorders often remain unrecognized, the study recruited non-treatment-seeking primary care patients at high risk for alcohol use disorders based on frequent heavy drinking (at least twice weekly, 4 or more drinks per day for women and 5 or more per day for men). Recruitment included a letter from the patient’s primary care clinician or the principal investigator of CHOICE if the clinician preferred. Eligible, consenting participants were randomized 1:1 to usual primary care plus the CHOICE intervention or usual primary care alone and all were assessed at baseline (in person), 3 months (telephone only) and 12 months.

A total of 304 patients were recruited. While results of the trial were not known at the time of the INEBRIA workshop in Stockholm and will be reported in another publication, recruitment and conduct of the trial yielded several useful qualitative observations. First, anecdotal reports from patients indicated that the process of recruitment alone—reaching out to primary care patients and assessing their drinking and related symptoms—resulted in decreased drinking or abstinence. In addition, nurses’ reports during supervision sessions seemed to show that patients’ stated readiness to change was variable over time and did not seem to predict whether or not the patient would change during the 12 months intervention. Some patients who said they would never change their drinking—because it was a central part of their lives and personal satisfaction—stopped drinking unexpectedly during the 12 months intervention, whereas some who indicated they wanted to make a change never did so during the 12 months.

These qualitative observations will deserve exploration after the main trial is analyzed, as they may have important implications for practice and research. First, these preliminary, anecdotal reports seem to support Orford and colleagues’ model of a “Catalyst System” that leads people with alcohol use disorders to decide to change their drinking [90, 91]. It may be that collaborative care visits—repeated so-called “brief interventions” and a nonjudgmental, ongoing relationship—function in part as a catalyst system along with other experiences patients are having outside the health system, to move patients toward change. Potentially, the training of primary care clinicians could emphasize basic activities such as ongoing assessment and proactive outreach in primary care that may act as a catalyst for change. These basic elements should be studied in future research, and in particular, the element of proactive outreach deserves rigorous study as an active ingredient of primary care-based alcohol interventions. Second, nurses’ reports of the stated readiness to change of patients randomized to the CHOICE intervention supported a fluid concept of readiness to change. At any particular time, a patient’s articulation of readiness might best be thought of as the momentary balance of their fluctuating ambivalence towards their drinking and possibility of change, consistent with the literature. These qualitative findings suggest that primary care clinicians might be trained to focus on the potential benefit of repeated small discussions of drinking that can act as catalysts to influence patients’ ambivalence towards their drinking. This observation has implications for primary care practice: offer brief interventions irrespective of patient statements of readiness to change. Primary care providers and integrated behavioral health clinicians have often been trained to consider readiness to change, and some conclude that there is nothing to offer patients who say they are not “ready” to make a change. Clinicians must be taught that ambivalence is the norm, and that momentary statements by patients that they have no interest in considering changing their drinking do not imply there is no benefit of brief discussions about unhealthy alcohol use.

### Treatment of alcohol dependence in primary care: the 15-method

#### Sara Wallhed Finn, MSc, Ph.D. student

The majority of individuals with alcohol use disorders have a mild-to-moderate severity disorder [84, 92]. They have fewer comorbidities, a stable social situation, and lower rates of treatment compared to individuals with severe alcohol use disorder [93]. Offering effective treatments in primary care may be less stigmatizing than referrals to treatment in specialist care, and would reduce the number of patients who would be lost during the referral process. Thus, treatments in primary care could reach more people. Some data suggest that treatments delivered by primary care clinicians could be promising [94], and that many patients with alcohol use disorders may prefer to seek treatment in primary care [95]. However, primary care clinicians often struggle with heavy workloads and time constraints. New interventions that are intended to be delivered by primary care clinicians need to be brief and effective.

A stepped care model, which begins with brief interventions and continues to more extensive treatments only if needed, may be a cost effective approach to treating alcohol use disorders in primary care [96]. One promising approach to treating alcohol use disorders in primary care with a stepped care model is to deliver multiple sessions of BI with pharmacological treatment.

We developed the 15-method, a stepped care model for alcohol use disorders in primary care. The name “15-method” refers both to that the length of the sessions is 15 min, and that the target group for the interventions in the last two steps are patients scoring above 15 points on the Alcohol Use Disorders Identification Test (AUDIT) questionnaire [97]. The first of three steps is screening to identify patients with hazardous alcohol consumption and a brief intervention session for patients who screen positive [27]. Step two is an assessment focusing on the consequences of alcohol consumption, where the patient completes questionnaires, submits a blood sample for biomarkers, and then participates in a feedback session which lasts 30 min [98]. The third and last step is four sessions of cognitive-behavioral therapy and motivational interviewing, also known as guided self-change [99, 100]. Each session has a theme to facilitate behavior change: goal setting, self-monitoring of alcohol consumption, identifying risk situations and problem solving. These four sessions can be combined with pharmacological treatments including acamprosate, disulfiram, nalmefene or naltrexone.

Through a randomized controlled non-inferiority trial where we included 288 adults who met ICD-10 criteria for alcohol dependence, we sought to evaluate whether the 15-method in primary care is equally effective as treatment in specialty care. The participants were randomly assigned to treatment either at a primary care unit or at a specialized addiction clinic. Regular clinical general practitioners at 12 primary care units were trained for 8 h in the 15-method. In the study, the primary care treatment began on step two in the 15-method with the feedback session, and patients who requested more treatment were offered step three. Treatment as usual in the specialty clinic included pharmacological and/or psychosocial interventions. The primary outcome was change in weekly alcohol consumption per self-report at follow up compared to baseline. Secondary outcomes included heavy drinking days, severity of dependence, consequences of drinking, psychological health, quality of life, satisfaction with treatment, and alcohol biomarkers.

If the 15-method is shown to be effective it can be one model for treatment of alcohol use disorders in primary care, which can expand the concept of BI and offer an alternative approach to RT. This would mean a more active role for primary care in providing treatment for a broader range of severities of alcohol use disorders, where referral would take place only in the case of no improvement after the initial treatment and perhaps restricting RT to the most complicated cases. This calls for efforts to strengthen the competence in primary care to provide alcohol treatment. From a research perspective, more work is needed to evaluate the effectiveness and implementation of extended BI in primary care.

### The stigma of unhealthy alcohol use

Alcohol use disorders are highly stigmatized [101–104], and much data indicate that stigma undermines efforts to give and get care for unhealthy alcohol use [58, 105–111]. Stigma refers to prejudice, stereotyping, and discrimination from the general public towards people with alcohol use disorders, as well as the mechanisms through which these negative attitudes, beliefs, and actions negatively impact people with alcohol use disorders [73, 75, 112–114]. Stigma occurs on multiple levels—structural/societal (e.g., discriminatory laws, policies; social norms of inclusion and exclusion), interpersonal (e.g., negative interactions with others), and internalized (e.g., shame and other processes within the individual), with cascading interacting influences on one another. The interpersonal level, as informed by the societal level, is an arena in which stigma is often expressed or enacted. Clinicians often hold stigmatizing beliefs regarding patients with alcohol (and drug) use disorders, and these beliefs can have an impact on how they provide care [105–109]. Moreover, from the patient perspective, people with substance use disorders report that they experience stigma when obtaining care [111], and general population surveys show that people with alcohol use disorders often forgo treatment because of a fear of the social consequences, such as embarrassment [56, 110]. In light of these findings, presentations at this meeting synthesized direct accounts of stigma from two perspectives: clinicians and non-treatment-seeking individuals with alcohol use disorders recruited from the general population.

### Alcohol-related stigma as a barrier to alcohol treatment

#### Emily C. Williams, Ph.D., MPH

Because stigma may be a barrier to provision of evidence-based alcohol-related care, we sought to identify whether alcohol-related stigma was expressed during qualitative interviews, which were conducted with primary care clinicians from 5 Veterans Health Administration (VA) clinics. The interviews were conducted for the purpose of understanding barriers and facilitators to provision of pharmacotherapy for alcohol use disorders. Key contacts and snowball sampling were used to recruit primary care providers with prescribing privileges from 5 VA clinics. Twenty-four clinicians completed 20–30-min in-person semi-structured interviews focused on barriers and facilitators to provision of pharmacotherapy for alcohol use disorder. Interviews were recorded, transcribed and analyzed using template (also called content) analysis to identify barriers to provision of pharmacotherapy. Alcohol-related stigma emerged as a barrier in primary analyses; thus, secondary analysis was undertaken to more comprehensively code common expressions of alcohol-related stigma that were previously described in the literature [83, 115]. These included: perceptions of character flaw (e.g., laziness), perceptions regarding control of and culpability for disease (e.g., beliefs that patients are choosing their condition and can quit if they are willing to do the work); social distancing (e.g., someone else should care for this condition); and labelling language (e.g., “those people” and “alcoholics”).

In this small qualitative study, alcohol-related stigma was evident in primary care clinicians’ responses to questions regarding provision of an evidence-based treatment for alcohol use disorder. While generalizability of findings may be limited, they are supported by a larger literature suggesting substance use stigma is common and contributes to suboptimal care for patients [105–107]. Findings from the present and previous studies suggest that stigma-reduction interventions aimed at clinicians who treat patients with alcohol (and other drug) use disorders may be needed. Only a handful of stigma-reduction interventions aimed at clinicians have been tested to date [116]. Contact-based training (e.g., direct contact with members of a stigmatized population) and education programs that target medical students show promise [116]. To our knowledge, no stigma reduction interventions have been explicitly incorporated into implementation trials of screening and brief intervention or other forms of evidence-based care, and given the current findings, this is an area in need of further study.

### Drinkers’ attitudes towards treatment

#### Ann-Sofie Bakshi, Ph.D.

Stigma may also be a barrier to patients’ seeking treatment. To inform efforts to get more people with alcohol use disorders into treatment, we conducted a qualitative study to better understand how affected individuals perceive treatment and why they do and do not seek it. To collect data, we conducted focus groups and semi-structured interviews in a non-treatment-seeking sample of individuals with alcohol use disorders in Sweden [72].

We recruited informants through a market research company’s panel consisting of 115,000 people in Stockholm, Sweden. We randomly selected 16,895 individuals to complete the Alcohol Use Disorders Identification Test-Consumption instrument [9] and a structured interview to assess past-year DSM-IV alcohol dependence symptoms [5]. Participants were eligible if they were 18–65 years of age and had three or more dependence symptoms. Of those screened for eligibility, 812 met inclusion criteria, 248 agreed to be contacted, and 32 agreed to participate.

We conducted seven focus groups using an open-ended, semi structured interview guide covering themes as views on alcohol consumption, alcohol problems, dependence, and treatment. We asked participants to discuss the themes in a more general way. We invited focus group participants to complete individual interviews regarding the same themes to share personal experiences of alcohol consumption. Almost all (*n* = 31) agreed to participate. All sessions were recorded and transcribed verbatim and analyzed through thematic content analysis [117, 118].

Consistent with previous research [56, 119, 120], stigma emerged as a major barrier to treatment. Participants emphasized the importance of keeping up appearance and a need to hide their drinking. To seek treatment was seen as shameful and as a personal failure. Alcohol problems were strongly associated with social deprivation and were linked to images of a stereotypical “drunkard” such as “the old geezer on a bench”. To seek and undergo treatment would be detrimental for the self-esteem, and to be medically diagnosed as a person with alcohol problems would imply an identity change from well-adjusted citizen to a drunkard—the lowest of the low—thus, indicating a very high threshold to seek treatment.

Participants had scarce knowledge about treatment options, mainly mentioning treatments requiring lifelong abstinence, older medications such as disulfiram, and hospitalization in rehabilitation centers, creating another threshold to seek treatment as described in previous research [121]. Participants did not see these treatments as appealing.

The main conclusion of these results is that the health care system needs to address the problem of shame and stigma by systematically implementing stigma reducing strategies in the context of healthcare settings in order to lower the threshold to seek help. One such strategy could be to offer treatment outside of specialty clinics, which may be a relief to many; for example, providing addiction treatment within primary care. Unlike specialty treatment, primary care settings are not linked with shame and a stigmatized identity. Moreover, modalities allowing individuals to get help while maintaining anonymity may reduce experiences of stigma, such as phone counseling, internet counseling, and other treatments not requiring an in-person encounter. Offering a wider set of treatment alternatives could promote autonomy, such as treatments tailored to individual needs and concern, providing a choice among treatment options, and providing a choice among treatment goals between total abstinence and reduced or controlled drinking. There is also a need to improve the publics’ knowledge about alcohol addiction and available treatment. In summary, we recommend several strategies to reduce the impact of the stigma of alcohol use disorders on patient outcomes: integrating alcohol treatments into primary care, empowering patients by providing them with treatment options, and reducing stigma through activities aimed at increasing knowledge about unhealthy alcohol use and treatment, targeting clinicians and the general public.

### Context: How should we conceptualize alcohol use disorders in general health care?

Some of the reluctance among general practitioners to ask questions about alcohol use comes from the realization that some patients will report more severe problems with their drinking, which practitioners do not feel qualified to address. Especially if heavy drinking is conceptualized as addiction or dependence, general practitioners will consider these as complex disorders requiring specialist attention. To make matters worse, many patients refuse to be referred to addiction specialists since addiction and treatment for addiction is heavily stigmatized. On the other hand, without concepts like dependence or addiction it is difficult to understand the difficulty to cut down on drinking despite serious consequences. Whether using these terms is helpful in primary care was another topic for debate at this meeting.

### Heavy use over time as a replacement descriptor for addiction: aligning alcohol with blood pressure and sugar plasma levels

#### Antoni Gual, MD, Ph.D.

Addictive behaviors are stigmatizing [73], and individuals are often labeled as being or not being “addicts” [122]. In reality those behaviors lie within a continuum that moves from occasional use to very heavy regular use, but health professionals are trained to identify if the condition (addiction) is present or absent.

This dichotomous approach in the alcohol field may explain part of the low identification and intervention rates [123]. On one hand treatment of alcoholism can be seen as a responsibility of specialized centers and on the other hand, counseling of risky drinkers may be understood as a marginal preventive activity that is not within the top priorities.

This dichotomous approach does not match reality and is against normal practice in primary health care, where blood pressure and blood sugar levels are good examples of a continuum approach. In fact, the different criteria used in the definitions of addiction have “heavy use over time” (HUOT) as the major underlying risk factor [124]. HUOT is responsible for the changes in the brain of drinkers and is also responsible for intoxication, withdrawal and tolerance phenomena, all of them regarded as central to current definitions of alcohol use disorders. On top of that, HUOT is responsible for the main social consequences of alcohol and for the majority of the alcohol-attributable burden of disease and mortality.

The effect of prolonged heavy use on the brain appears to be at least largely overlapping if not identical with what is called ‘alcohol use disorder’, and it is no surprise that a very high correlation between HUOT and number of DSM diagnostic criteria for alcohol use disorder has been shown in a population-representative sample in the National Epidemiologic Survey on Alcohol and Related Conditions [125].

Shifting from addiction to HUOT may have several advantages concerning clinical practice in primary health care: measuring consumption is easier, can be automated, can be monitored as other chronic conditions and can be fed back with very little time lag and investment.

In summary, most of the signs and symptoms that have been attributed to alcohol use disorder are actually the consequences of heavy drinking. Even though it may be argued that HUOT does not take the impulsivity element of addiction into account [126], it should be noted that ‘impulsivity’ appears more as an explanation than as a description, which is what HUOT intends to be [125]. Thus, the term “alcohol use disorder” is redundant and the term “heavy use over time” is all that is needed at a primary health care level. It is also a descriptor that fits better with the clinical routines of primary health care than the diagnostic criteria for alcohol use disorders, and in fact is not a new strategy: back in 1986 the Royal College of General Practitioners of London already proposed to treat alcohol problems following the model of hypertension [127]. In order to move in this direction, we need more research to operationalize the HUOT construct, and randomized controlled trials to show if the use of HUOT may lead to increased rates of identification of unhealthy alcohol use.

### “Addiction” is better than “heavy use over time” for responding to alcohol problems in primary care

#### Nick Heather, Ph.D.

Rehm et al. [124] have argued that substance use disorders, including alcohol use disorders, should be defined as ‘heavy use over time’ and that concepts of ‘dependence’ or ‘addiction’ are unnecessary. This misses the point that the patient’s problem is not heavy use over time but addiction to heavy use [126]. If heavy use over time fully described the problem patients and clinicians were confronted with, patients would cut down simply on learning that their alcohol use was harmful; the fact that they typically do not is sufficient to demonstrate that the concept of addiction is essential to understand and respond to patients’ harmful alcohol consumption.

Rehm and colleagues’ arguments are directed against an all-or-none, tick-box approach to defining dependence/addiction, as exemplified by DSM or ICD classifications. I fully agree that an all-or-nothing approach to alcohol use disorders is unhelpful in primary care but suggest that it is a continuous concept of addiction that is required, where addiction is seen as continuously distributed throughout the population of regular drinkers. Also, in a radical reframing of the concept, addiction can be characterized as persistent repeated failures to cut down or abstain from (alcohol) use despite prior resolutions to do so [128], a view of addiction that would fit the experience of primary care practitioners and make sense to them. Primary care interventions related to alcohol use may be of several different kinds but here there is a specific concern with the attempt to change the patient’s drinking behavior as part of health behavior change more generally. More precisely, I’m here concerned with the attempt to help patients desist from harmful behaviors rather than the equally important task of persuading them to take up new and beneficial behaviors.

How does this view of addiction contrast with the standard, ‘official’ view based on neuropsychiatric evidence that addiction is due to changes in the neurophysiology of reward and motivational pathways? First, the changes in the neurophysiology that are thought to underlie substance addictions have also been hypothesized to underlie so-called behavioral addictions, so that, for example, persistently repeated unhealthy eating, including repetitive late-night snacking, might also be partly determined by such changes. Secondly, whether or not this is true, my definition of addiction as persistently repeated breakdowns in resolutions to change behavior subsumes unhealthy behaviors that may be due to changes in brain functioning and those that may not be. Even where brain changes are involved, relapse to addictive behavior is not inevitable and thus the practitioner’s efforts to assist behavior change are still called for.

Rehm et al. also suggest that seeing alcohol use disorder as heavy use over time would reduce the stigma associated with alcohol problems. However, stigma, as an informal means of social control, is applied to any behavior that transgresses accepted norms and would continue to be applied to heavy use over time. And stigma arises not merely from verbal labels but from the act of publicly marking people out for special attention and discrimination from the majority, for example by inviting people to attend an appointment in a designated location for counselling or treatment in relation to an identified behavioral problem.

In any event, I do not suggest that primary care patients should be labelled as “addicts”; rather, addictive behavior in the primary care setting might usefully be called ‘hard-to-reduce or –eliminate behavior’ [129] and interventions could be conceptualized and taught as methods to help maintain health behavior changes that the patient has accepted are necessary [130]. There is clearly a range of factors that affect the patient’s ability to maintain desirable changes in behavior, including homelessness, food insecurity, domestic abuse, child care demands, employment opportunities, and psychiatric illness, and these clearly call for different types of intervention. However, an emphasis on the relapsing nature of persistently harmful drinking as a form of addictive behavior and as part of the problem of health behavior change in general, would be readily understood and accepted by primary care practitioners.

I have had many conversations with general medical practitioners and other primary care workers who say that, when the harmful nature of their behavior is pointed out to them, patients are typically quite sincere in their stated resolutions to cut down or quit drinking or to desist from whatever unhealthy behavior is in question but yet, in a majority of cases, fail to live up to their resolutions. It is this that leads practitioners to be pessimistic about the effectiveness, for example, of brief interventions aimed at reducing hazardous or harmful drinking. My central point here is that, if practitioners were encouraged to see such failures in sincerely-made resolutions as examples of addictive behavior, as I contend that they should be, and to see the problem as one of helping the patient in the difficult task of maintaining changes in addictive behavior and preventing relapse to unhealthy behavior, this would accord usefully with their clinical experience and, moreover, would suggest ways of helping patients that have been developed to prevent relapse in formal treatment of addictive behaviors.

### Context: how can we design SBI to improve global public health?

SBI makes good intuitive sense. Most of the published research in this field comes from high income countries where many attempts have been made to implement this strategy. As has been described in this report, implementation of SBIRT in healthcare settings remains a challenge. An even larger challenge is implementation in low and middle income countries. Despite the limitations of SBI acknowledged above, in many cases, where specialized addiction services are scarce or non-existent, SBI in primary care is the only possible strategy to manage alcohol problems. Two speakers at the meeting widened this discussion to a more global perspective.

### Perspectives from Latin America

#### Marcela Tiburcio, Ph.D.

The burden that alcohol consumption inflicts on the population’s health in Latin America is well documented. There is limited information about the capacity of the countries in the Region to offer prevention and treatment services for people at risk. However, it is known that the healthcare systems are fragile and insufficient, and there is lack of human and financial resources for research and assessment of substance use disorder treatment programs [131, 132].

Due to the need to identify individuals at risk and to prevent the negative consequences of harmful and hazardous drinking with limited resources, some countries of the Region have turned to SBIRT as a reasonable option to deal with this public health problem. While the level of progress varies from one country to another, significant advances have been made overall. For instance, in Central America the World Health Organization (WHO) Mental Health Gap Action Programme is being adapted to treat mental health conditions including substance use disorders [131].

Chile [133, 134], Colombia [135, 136], and Panama [137] have developed national plans and clinical guidelines to incorporate the AUDIT and the Alcohol, Smoking and Substance Involvement Screening Test (ASSIST) packages into primary healthcare; the implementation of such programs now represents a great opportunity not only to assess the results of SBIRT in reducing alcohol consumption, but also to explore the barriers and facilitators at the organizational and individual level.

Different actions are taking place in the Region to disseminate SBIRT procedures. In Brazil, there are different experiences of training health professionals working primary healthcare facilities to use SBI as a routine practice both in face-to-face [138, 139] and e-learning modalities [140, 141] with good results. In other countries, the training initiatives use e-learning courses developed by the Pan American Health Organization [142] as support material [132].

As to research, pregnant women constitute a particularly noteworthy target population in Argentina where there is a research group interested in developing and testing programs based on WHO and National Institute on Alcohol Abuse and Alcoholism recommendations to detect women at risk and to promote abstinence [143]. In Colombia, high school and university students are the focus of different research projects aimed to develop and assess indicated and selective prevention programs including early detection of at-risk students.

In Mexico, there are over 400 prevention and treatment centers nationwide which offer free or low cost services based on brief interventions; however, the efficacy of their programs has not been fully established yet. In addition, about 30% of primary healthcare facilities have protocols to detect and treat mental health conditions including substance use disorders [144]. There is some progress in training healthcare professionals to administer the ASSIST at tertiary health care facilities. Regarding referral to treatment, there is a shortage of government-funded options for alcohol dependent individuals; the majority of the available options are 12-step residential programs and Non-Governmental Organizations.

In summary, there are many efforts in Latin America to disseminate SBIRT, but evidence about its efficacy and effectiveness are still needed; thus, the main recommendation is to fund and encourage research, particularly implementation science and evaluation of programs already being implemented, to generate the required data that could inform the policy making process.

### Perspectives from India

#### Vivek Benegal, MD

While traditionally designated as a predominantly abstinent culture, levels of consumption of alcohol and the levels of alcohol-related harm to self and to other-than-the-drinker, in India, have been rising significantly. It is estimated that the per capita consumption of alcohol in India increased by 55% in the 20-year period from 1992 to 2012, with a sharp increase in risky drinking behaviors especially among young people and women [145]. This is reflected in a 66% rise in recorded alcohol-related deaths in the ten years from 2003 to 2013 [146]. Previous studies have documented that more than a third of the patients in medical wards of hospitals and over 60% of injuries in the emergency rooms are associated with harmful alcohol use [147]. It is expected that early detection in various health settings, starting from general healthcare, with brief interventions and referral to treatment within a stepped care format of escalating complexity would thus be the natural model of treatment to be adopted. Not so.

The popular discourse in India has found it difficult to shift from the Temperance view of alcohol and this is reflected in the political fascination with total Prohibition as the favored means of alcohol control [148, 149]. Since excise duties from alcohol represent more than 20% of the earnings of most states in the country the fiscal and other consequences of Prohibition, most often make the exercise fairly short-lived. The focus of alcohol control has centered around the dependent drinker or “addict” and the funding of a few abstinence based de-addiction centers and rehab centers. It has also resulted in the mushrooming of boot-camp style “rehab” centers which are merely convenient centers of incarceration. Despite adequate evidence the focus has not been on the larger spectrum of consequences from the harmful use of alcohol, on disease burden, in the causation of the major non-communicable diseases, its role in the link with non-communicable diseases (NCDs) and on social cost [150, 151].

In this context, there have been a few studies of SBIRT in India, including one randomized controlled trial (unpublished dissertation) [152–154]. Most have demonstrated efficacy in the research situation. However there have been no large scale studies or the deployment of SBIRT in the community treatment settings. Part of the problem is the lack of a stepped care system and the lack of trained personnel to run the SBIRT processes [147]. There are several ongoing attempts to include Early Detection and Brief Interventions into the treatment pipelines of primary healthcare personnel. The Government of India has created a cadre of doctors and counsellors to be engaged primarily in interventions for NCDs. Alcohol is one of the five major preventable causes of NCDs, and the efforts have been to include SBIRT for alcohol and tobacco in the treatment as usual of NCDs.

Several recommendations for practice and policy are as follows. There is thus a need to create resources for training, monitoring and hand-holding of the trained personnel. Reframing alcohol misuse as a preventable health risk, rather than a characterological frailty and pairing alcohol enquiries with general health enquiries, will, it is hoped, reduce the stigma and raise awareness for the need for alcohol controls. There is also a need to utilize newer evidence based practices—including pharmacological interventions and anti-craving agents, brief medicine management—along with the motivational interviewing strategies and the other approaches to behavior modification. Given the pressures and the heavy workload of the health personnel in India, this will not be easy.

## Conclusions

It is time to rethink alcohol SBI in primary care [155]. Serious concerns that well-designed studies find effects only on consumption and not other important outcomes suggest that it is time for large controlled studies to test efficacy for improving health. We need to understand more about what works best in SBI both to make it as effective as possible, and to know what is essential for successful implementation. Stigma appears to be a barrier to provision and receipt of care; it needs to be addressed to be able to get help to those who need it; research will help delineate how best to address it. Likely public attitudes as well as those of health professionals need to change. Making anonymous options available may help. Providing multiple evidence-based treatment options in general healthcare settings and specialty settings will likely help. SBI targets unhealthy alcohol use which can be tracked as heavy drinking occasions. That can be the target behavior. Yet it should not be forgotten that for many this is an addictive behavior so that attention needs to be given to how to change a difficult-to-change behavior. Addressing dependence (moderate to severe alcohol use disorder) has been given too little attention in SBI; clinical and research attention needs to be given to better connecting people to needed care, and to not relying on referral as the only solution. Treatment for alcohol use disorders should begin in general health settings; models for such care should be developed and studied. From an implementation perspective, the challenges present in varied practice settings and countries should be given attention in research and practice. Clinicians should identify unhealthy alcohol use in general health settings, and should give relevant counsel. BI likely needs to be repeated, and conducted in the context of longitudinal care, in order to be effective. Stepped care, as is done for many other health conditions, shows promise (repeated counseling, pharmacotherapy, psychosocial treatments, mutual help and self-help, depending on need and readiness).

## Abbreviations

INEBRIA: International Network on Brief Interventions for Alcohol & Other Drugs
SBI: Screening and brief intervention
SBIRT: Screening, brief intervention, and referral to treatment
RT: Referral to treatment
BI: Brief intervention
CHOICE: Choosing Healthier drinking Options In primary CarE
VA: U.S. Department of Veterans Affairs
AUDIT: Alcohol Use Disorders Identification Test
HUOT: Heavy use over time
ICD: International Classification of Diseases
DSM: Diagnostic and Statistical Manual of Mental Disorders
WHO: World Health Organization
ASSIST: Alcohol, Smoking and Substance Involvement Screening Test
NCD: Non-communicable diseases
